# Human targeted phenobarbital presents a poor substrate of gut microbiome deciphering new drug targets beyond pharmacokinetic curbs

**DOI:** 10.1186/s40360-022-00618-x

**Published:** 2022-11-14

**Authors:** Imran Mukhtar, Haseeb Anwar, Arslan Iftikhar, Heba E. Hashem, Qasim Ali, Farhan Siddique

**Affiliations:** 1 Department of Physiology, Faculty of Life Sciences, Government College University, Faisalabad, Pakistan; 2 Department of Chemistry, Faculty of Women, Ain Shams University, Heliopolis, 11757 Cairo Egypt; 3 Department of Botany, Government College University, Faisalabad, Pakistan; 4 School of Pharmaceutical Science and Technology, Tianjin University, Tianjin, 300072 People’s Republic of China; 5 Department of Pharmaceutical Chemistry, Faculty of Pharmacy, Bahauddian Zakariya University, 60800 Multan, Pakistan

**Keywords:** Microbiome, Microbial lysate, Phenobarbital, Pharmacokinetics

## Abstract

**Background:**

The gut microbiome, a new organ of the body, can potentially alter the pharmacokinetics of orally administered drugs through microbial enzymes. However, absorption of orally administered non-antibiotic drugs by the gut microbiome, during drug-microbiome interaction, is barely addressed. Structural homology studies confirm similar membrane transport proteins in gut epithelial cells and the gut microbiome of the host that may compete for drug substrates with the host itself for its absorbance. Therefore, it is hypothesized that orally administered human targeted phenobarbital may interact and/or be uptake by the gut microbiome during its transit through the small intestine.

**Methods:**

In the current in vivo study, thirty-six male *Wistar albino* rats were divided into six groups including one control and 5 treatment groups, each having an equal number of rats (*n* = 6). Phenobarbital was administered orally (single dose of 15 mg/kg bw) to treatment groups. Animals were subsequently sacrificed to harvest microbial mass pallets residing in the small intestine after 2, 3, 4, 5, and 6 h of phenobarbital administration. Phenobarbital absorbance by the microbiome in the microbial lysate was estimated through RP-HPLC–UV at a wavelength of 207 nm.

**Results:**

Maximum phenobarbital absorbance (149.0 ± 5.93 µg) and drug absorbance per milligram of microbial mass (1.19 ± 0.05 µg) were found significantly higher at 4 h of post-administration in comparison to other groups. Percent dose recovery of phenobarbital was 5.73 ± 0.19% at 4 h while the maximum intestinal transit time was 5 h till the drug was absorbed by the microbes. Such results pronounce the idea of the existence of structural homology between membrane transporters of the gut microbiome and intestinal enterocytes of the host that may competitively absorb orally administered phenobarbital during transit in the small intestine. The docking studies revealed that the phenobarbital is a poor substrate for the gut microbiome.

**Conclusion:**

Gut microbiome may competitively absorb the non-antibiotics such as phenobarbital as novel substrates due to the presence of structurally homologous transporting proteins as in enterocytes. This phenomenon suggests the microbiome as a potential candidate that can significantly alter the pharmacokinetics of drugs.

## Background

Currently, the microbiome is being investigated effectively for its various functions, without which the human body is unable to compensate for its normal functions [1]. The symbiotic relationship of the normal microbiome with the host is extended to various inhabiting species. Collectively, this host-microbiome interaction can harbor trillions of cells, more than 100 folds of genes present in the human genome. Such a mixture of human and microbial cells defines the human body as a supra-organism [2, 3]. The microbiome is a blend of both gram-positive and gram-negative microbes, contributing in different dimensions to maintain the active status of host health [4]. Diversity and richness of microbiome vary from proximal to distal part along the length of the gastrointestinal tract [5]. However, more than 90% of the microbiome comprises of Firmicute and Bacteroidetes phylum [6] along with other bacterial species [7].

In the gastrointestinal tract, the complex micro-environment is highly diversified not only by anatomically but also physiologically and biochemically which can influence dissolution, stability, pre-systemic elimination, and absorption of a variety of drugs [8]. The small intestine, a major absorptive site in the body for nutrients and drugs, harbors diversified microbes belonging to *Streptococcaceae*, *Bacilli*, *Actinomycinacae,* and some members of phylum Actinobacteria inside the lumen, embedded or attached in the mucus layer and the intestinal crypt [9]. Previous insights focused on absorption of drugs largely through the duodenal portion of the intestine while the influence of inhabiting microbes got less attention [10]. Nevertheless, pertinent functions of various drugs need more attention on a pharmacological scale to minimize this critical concern [11, 12]. However, drug-microbes interactions fared better as a complement to the efficacy and toxicity of drugs [13]. Currently, microbial encoded enzymes present drug targets to alter the pharmacokinetics and pharmacodynamics of orally administered drugs [14].

Different transporting proteins have commonly evolved to mediate nutrient absorbance in all forms of life. Among them, proton-dependent oligopeptide transporters (POT’s) for example hPepT-1 (human peptide transporter-1) in humans [15] and YdgR (dipeptide and tripeptide permease A) in bacteria [16] mediate absorbance of di and tri peptide from dietary nutrients [17] and drug molecules [18]. hPept-1, found in the apical membrane of the enterocyte, is a high capacity and low-affinity transporter [19] analog to YdgR found in the inner membrane of the microbial cell. Both share 52% homology in the conserved amino acid sequence [16]. Moreover, YdgR’s orthologs: YjdL, and YbgH had shown more affinity towards dipeptide regarding substrate preference [20] and 50% homology in the conserved amino acid sequences [21].

Drugs like sulpiride, amoxicillin, levodopa, and oseltamivir have been proposed as substrates of YdgR in in vitro trials [22]. Recently, paracetamol had been declared as a substrate of the gut microbiome, as a varying amount of orally administered paracetamol was detected at different transit time intervals. Another study from our group has provided evidence of competitive absorbance of paracetamol and sulpiride by epithelial cells and microbiome due to the co-existence of similar transport systems for the same substrate [23, 24].

**Fig. 1:**
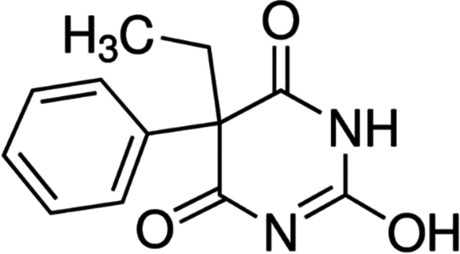
Chemical structure of phenobarbital

Traditionally, preclinical trials evaluating the pharmacokinetic parameters of new chemical entities (NCE’s), usually ignore the direct absorption of drugs by the gut microbiome. If this area is addressed properly, it may unfold novel strategies to improve the efficacy and to minimize the plethora of toxicity confronted during the routine pharmacokinetic curbs. By extending the empirical link with previous efforts, we selected the non-antibiotic phenobarbital (Fig. 1), prescribed worldwide as a drug of choice in neurological disorders like epilepsy, to check if the gut microbiome is a druggable target and whether phenobarbital is directly absorbed by the microbiome.

## Methods

### Animals

Wistar albino (*n* = 36) male healthy adult rats having age (8–10 weeks) and average weight (160 ± 20gm) were selected randomly from the Animal Facility of the Department of Physiology, Government College University, Faisalabad. Animals were raised in strictly hygienic, controlled temperature (25 ± 2 °C), humidity (40–60%), and light–dark cycle (12 h each) conditions. The animals were given free access to a standard chow maintenance diet (CMD) and water ad libitum. Animals were acclimatized for 7 days before starting the study. Animals were kept feed restricted 8 h before administration of phenobarbital, keeping in view the guidelines of the ERB (Ethical Review Board) Ref. No. GCUF/ERB/131, Government College University Faisalabad, Pakistan. Animals were randomly divided into six groups including control as an untreated group (A1) and phenobarbital-treated groups; A2, A3, A4, A5, and A6, each group having the same number of rats (*n* = 6). Animals of phenobarbital-treated groups were administered with a single dose of phenobarbital 15 mg/kg bw [25], through an oral gavage rat feeding tube (16–18 gauge, 0.79–1.18 cm in length), afterward permitting the free access to feed. Animals of treated groups were sacrificed adapting the procedure of decapitation, 2, 3, 4, 5, and 6 h after administration of phenobarbital.

### Isolation of the microbial mass and preparation of microbial lysate

Microbial mass pallet was harvested from digesta of the small intestine as described previously [23]. The microbial mass pallet was re-dissolved in 1 mL of acetonitrile (ACN) and stayed overnight to ensure lysis of all microbial cells. The lysed solution was vortexed, and the supernatant was evaporated through nitrogen gas to obtain the dried residual microbial lysate. The microbial lysate was dissolved in 800 µL of desired mobile phase and filtered through the nylon syringe filters having a pore size of 0.45 µm (Milli Pore®, USA) and preserved at -20℃ till injecting the HPLC system for the detection of unknown concentration of phenobarbital in lysate solution.

### HPLC system and conditions

**Fig. 2:**
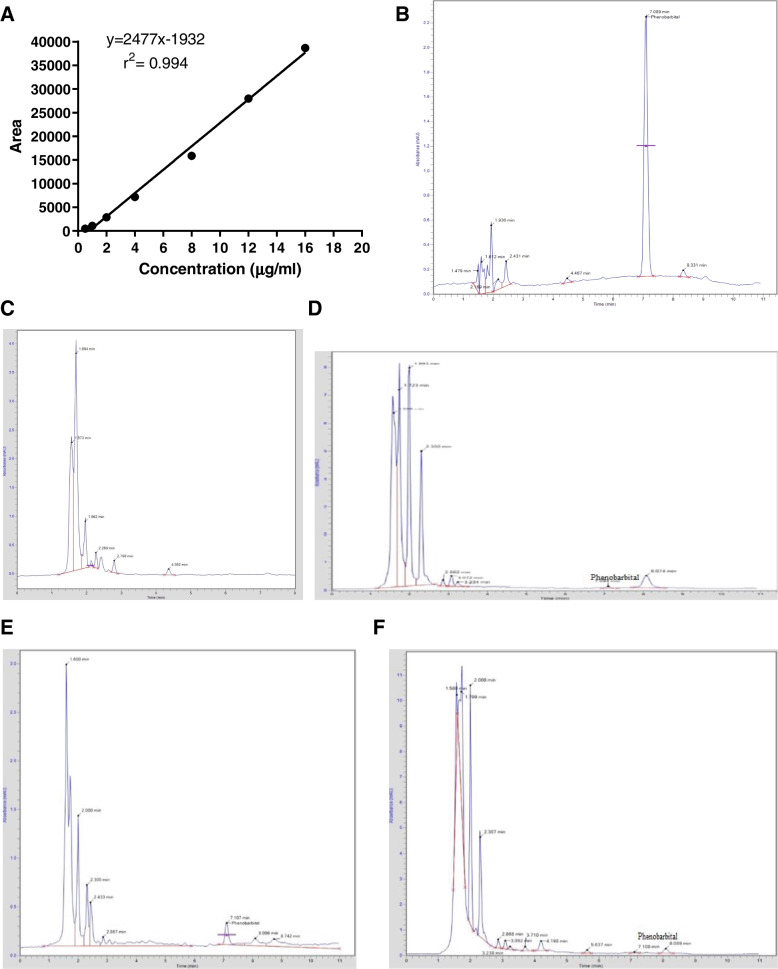
**A** Phenobarbital calibration curve (0.5–16 µg/mL) studied. **B** Chromatogram of phenobarbital standard solutions (12 µg/mL). **C** Chromatogram of an untreated sample. **D** Chromatogram of the phenobarbital-treated sample at 3 h. **E** Chromatogram of the phenobarbital-treated sample at 4 h. **F** Chromatogram of the phenobarbital-treated sample at 5 h post-phenobarbital oral treatment (15 mg/kg of BW) sampling time

Phenobarbital-treated and untreated microbial lysate samples were analyzed through the HPLC system, as described by [26] with slight modifications. The mobile phase consisting of acetonitrile (ACN) and water (25:75 v/v) was maintained at pH (2.5) by the addition of H_3_PO_3_ solution. HPLC system (Perkin Elmer®, USA) consisting of C18 column (5 µm, 250 × 4.6 mm) attached with UV/VIS Detector (Shelton CT®, 06,484 USA). The oven temperature was maintained at 30 °C. Chromera® software (Version. 4.1.2.6410) was employed to quantify phenobarbital concentration in the final samples. A volume of 10µL was injected by syringe, maintaining a constant flow rate of 1.5 mL/min to find the unknown phenobarbital concentration in each sample in comparison to the calibration curve (Fig. 2A) with regression equation (Y = 2477x-1932) calculated against different (7-point) phenobarbital standards (0.5, 1, 2, 4, 8, 12, 16 µg/mL) at given HPLC conditions with referenced wavelength at 207 nm showing retention time of 7.08 ± 0.3 min. Representative chromatograms for phenobarbital standard solution; 12 µg/mL (Fig. 2B), blank untreated sample (Fig. 2C)**,** phenobarbital-treated samples at 3, 4, and 5 h transit time are shown in (Fig. 2D, E & F respectively). The Correlation Coefficient (R^2^) was 0.994 while the percentage recovery was 93.87%.

### Molecular modeling study

A molecular docking study was performed using MOE (Molecular Operating Environment) software 10.2008 [27] to evaluate the interaction of phenobarbital with the gut microbiome and explain how gut microflora influences phenobarbital absorption and its availability in the gut. Docking study of phenobarbital shed light on its potential binding modes with transport protein *E. coli* BL21(DE3) and membrane protein *E. coli K-12, Lama glama*, (A) (PDB ID: 5A9D) and (B) PDB ID: 6GS4), respectively. The protein data bank files (PDB: 5A9D and 6GS4) were downloaded and prepared for molecular docking by 3D protonation, fixed potential, energy minimization, and prediction of the most active site keeping the default parameter.

### Statistical analysis

Different data sets produced were analyzed statistically by applying ANOVA (one-way analysis of variance) by GraphPad Prism 6.0 San Diego, CA 92,108, USA. Post hoc test, DMR (Duncan Multiple Range test) was employed to determine the significance among various groups through software Costat version 6.4.by setting (*P* ≤ 0.05).

## Results

**Fig. 3:**
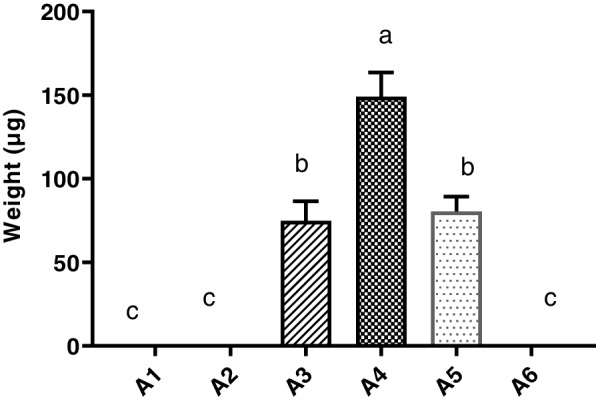
Total phenobarbital absorbance (µg ± SE) by the whole small intestine microbiome determined in various groups: A1 = Control (untreated), drug-treated groups based upon post-phenobarbital oral treatment (15 mg/kg of body weight) at sampling times A2 = 2 h, A3 = 3 h, A4 = 4 h, A5 = 5 h and A6 = 6 h. Alphabets on mean bars show a significant difference between groups (*P* ≤ 0.05)

**Fig. 4:**
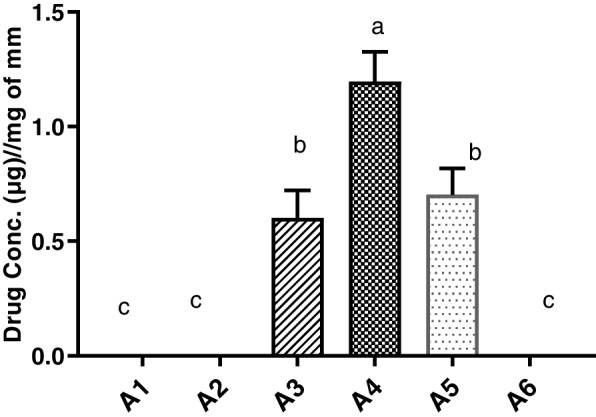
Phenobarbital absorbance (µg ± SE, *N* = 6) per mg of microbial mass measured in different groups: A1 = Control (untreated), groups based upon post-phenobarbital oral treatment (15 mg/kg of body weight) at sampling times A2 = 2 h, A3 = 3 h, A4 = 4 h, A5 = 5 h and A6 = 6 h. Alphabets on mean bars show a significant difference between groups (*P* ≤ 0.05)

**Fig. 5:**
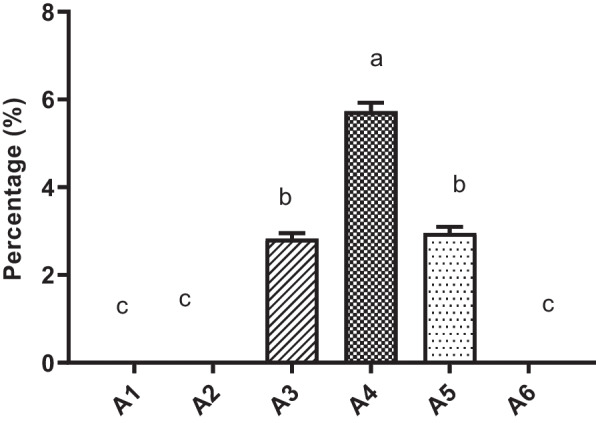
Percentage dose recovery (% ± SE, *N* = 6) from administered dose of phenobarbital determined in various groups: A1 = Control, drug-treated groups based upon post-phenobarbital oral treatment (15 mg/kg of body weight) at sampling times A2 = 2 h, A3 = 3 h, A4 = 4 h, A5 = 5 h and A6 = 6 h. Alphabets on mean bars show a significant difference between groups (*P* ≤ 0.05)

Phenobarbital absorbance (µg) by the gut microbiome was explicitly observed in groups A3, A4, and A5 at 3, 4, and 5 h intestinal transit time, respectively. Drug absorbance was initially observed in samples after 3 h post-drug administration and continued until 5 h post-drug administration transit time. Total phenobarbital absorbance by the gut microbiome was found significantly higher (*P* ≤ 0.05) in group A4 (4 h) post-drug administration transit time as compared to the remaining groups (Fig. 3). Drug absorbance by the gut microbiome was also observed in groups A3 and A5 significantly higher (*P* ≤ 0.05) as compared to the control group, A2 and A6, but significantly (*P* ≤ 0.05) low as compared to A4. Maximum phenobarbital absorbance (µg) per mg of microbial mass (Fig. 4) was found significantly higher (*P* ≤ 0.05) in group A4 as compared to other groups. Percent dose recovery was significantly higher (*P* ≤ 0.05) in group A4 (4 h) post-drug administration as compared to groups A3 and A5 at 3 and 5 h post-drug administration (Fig. 5). Maximum phenobarbital absorbance was observed in group A4 at 4 h post-drug administration, while maximum post-drug administration time was 5 h at which phenobarbital was detected. Phenobarbital was not detected at 6 h post-drug administration time. Phenobarbital was not detected in the gut microbiome of group A1 (control), A2, and A6 at 0, 2, and 6 h post-drug administration.

**Fig. 6:**
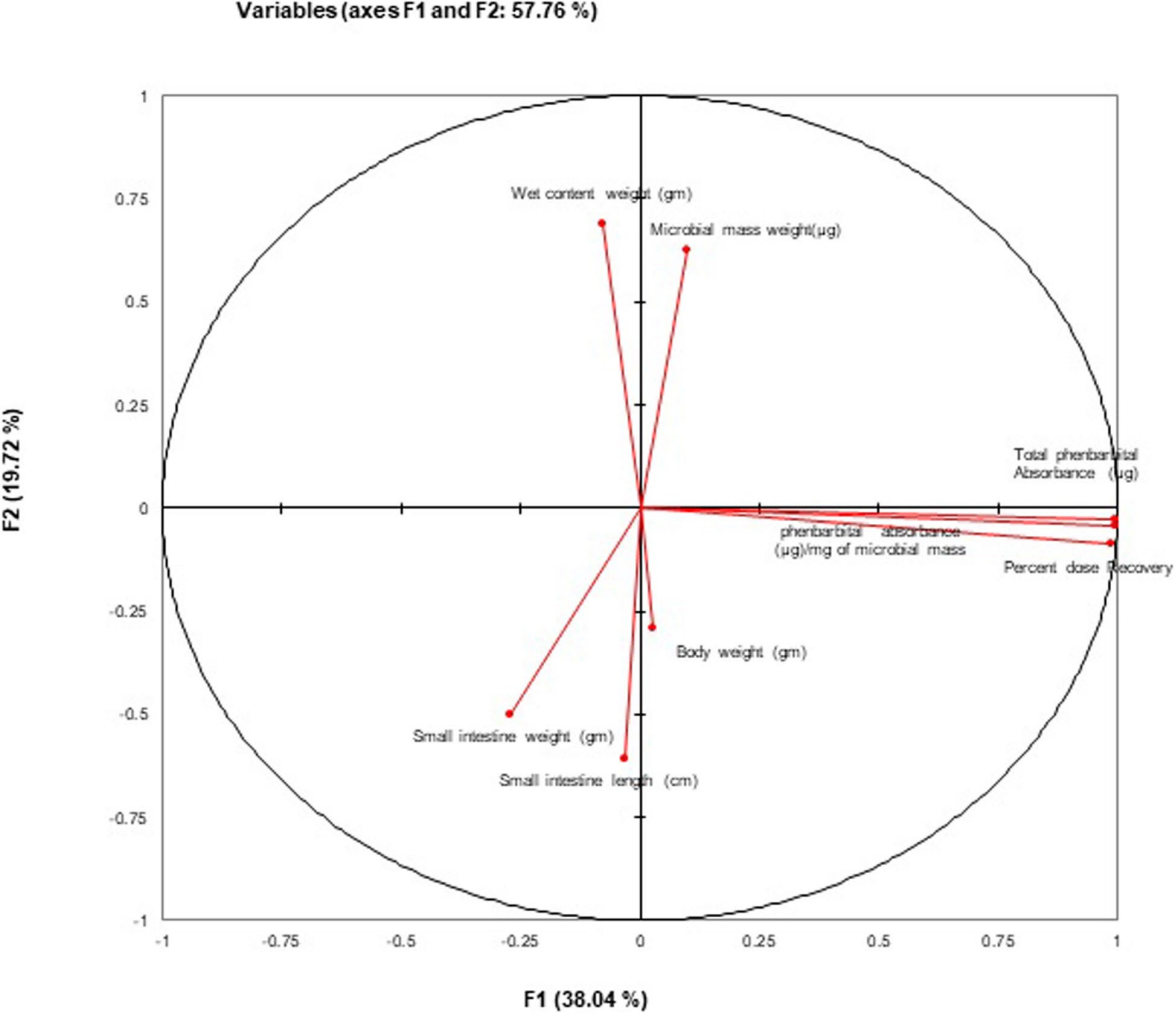
Principal component analysis (PCA) of different attributes observed for post phenobarbital oral treatment (15 mg/kg of body weight) at different transit times in the small intestine

Total body weight, small intestine length, the weight of small intestine, wet content weight (and total microbial mass exhibited non-significant difference (*P* ≥ 0.05) among control group and phenobarbital-treated groups Table 1. Different attributes measured during the study indicate a significant contribution of components (F1 and F2) during principal component analysis (PCA). The contribution of component F1 (38.04%) was higher as compared to the F2 component (19.72%) shown in (Fig. 6). Total Phenobarbital absorbance by whole microbes residing in the gut show a significant positive correlation with drug absorption per mg of harvested microbial mass (0.989***) and administered dose of Phenobarbital (0.992***).

**Table 1:** Various physical parameters of control and phenobarbital treated groups

Groups (Transit Time)	Body Weight (g)	Small Intestine Length (cm)	Small Intestine Weight (g)	Wet Content Weight (g)	Microbial Mass (mg)
A1 (control)	171 ± 3.29	104.8 ± 1.60	5.75 ± 0.35	2.18 ± 0.32	128.3 ± 7.44
A2 (2 h)	176 ± 5.28	108.1 ± 0.90	5.67 ± 0.37	2.64 ± 0.17	118.5 ± 11.42
A3 (3 h)	175 ± 4.62	104.8 ± 1.37	5.95 ± 0.30	2.23 ± 0.18	125.3 ± 5.67
A4 (4 h)	173 ± 7.90	106 ± 0.93	5.30 ± 0.26	2.52 ± 0.25	125.5 ± 5.47
A5 (5 h)	181 ± 3.14	108.1 ± 0.87	5.19 ± 0.35	2.45 ± 0.24	116.6 ± 7.40
A6 (6 h)	168 ± 2.84	106.6 ± 1.05	5.86 ± 0.40	2.36 ± 0.29	118.8 ± 5.48

**Fig. 7:**
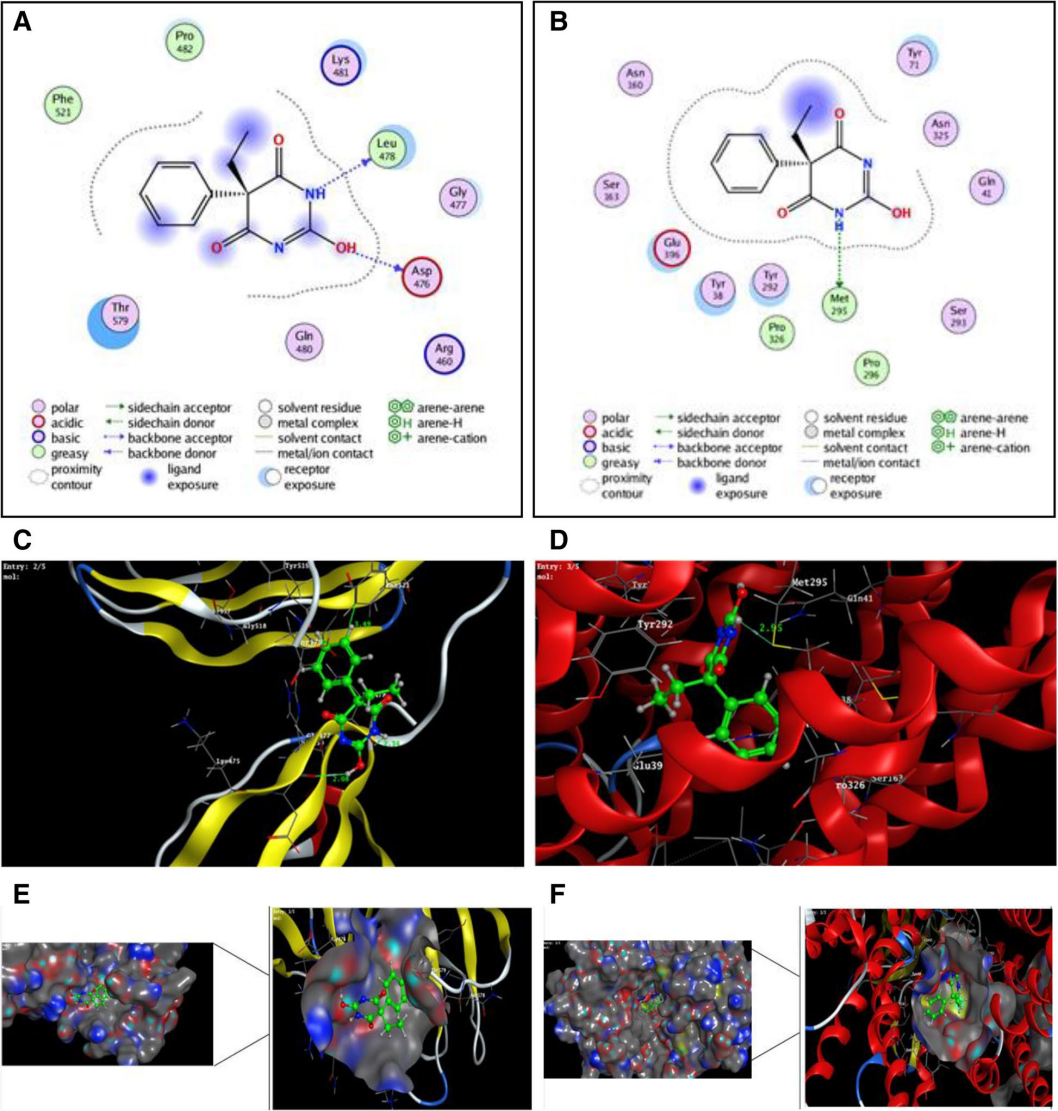
Two-dimensional interaction diagrams of phenobarbital docked in the active sites of (**A**) transport protein Escherichia coli BL21(DE3) (PDB ID: 5A9D) and (**B**) membrane protein *Escherichia coli K-12*, Lama glama (PDB ID: 6GS4). Three-dimensional interaction diagrams of phenobarbital docked in the active sites of (**C**) transport protein Escherichia coli BL21(DE3) (PDB ID: 5A9D) and (**D**) membrane protein *Escherichia coli K-12*, Lama glama (PDB ID: 6GS4). Surface binding pose of phenobarbital in the active site of protein (**E**) PDB ID: 5A9D and (**F**) PDB ID: 6GS4; with conformational behavior within the surface-active site of target proteins

According to the docking simulation result observed in (Fig. 7) phenobarbital was embedded poorly within the active pocket of target proteins (PDB ID: 5A9D) (A) and (PDB ID: 6GS4) (B) with a docking score 4.9 and 5.5, respectively. And root mean standard deviation (RMSD) = 0.90 and 1.66 Å, respectively. Phenobarbital fixed within the active site of transport protein (A) by two hydrogen bonds with the essential amino acids Leu478 and Asp476 (distance: 2.34 and 2.08 Å, respectively) and arene-cation interaction with Phe521 (3.49 Å) (Table 2). On the other hand, NH of phenobarbital molecule allowed only one hydrogen bond donor with the side chain of Met295 (distance: 2.95 Å) within the binding pocket of membrane protein (B) (Fig. 7A & B).

**Table 2:** Protein–ligand interaction results

Protein	Docking score	Active Amino acid residue	Type of interaction	Distance Å
Transport protein (A)	4.9	Leu478 Asp476 Phe521	H-donor H-donor Ionic	2.34 2.08 3.49
Membrane protein (B)	5.5	Met295	H-donor	2.95

## Discussion

The gut microbiome has been studied extensively with its active role in the biotransformation of various orally administered drugs. The established flora in the gut influences pharmacokinetics parameters such as drug absorption and bioavailability [14]. It was also observed that as the drugs stay for longer periods in the gut, therefore, their biotransformation is more rapid [28, 29]. In the current in vivo trial, we assessed the competitive absorbance of orally administered phenobarbital by the gut and residing microbiome in an experimental rat model. Physical parameters such as body weights, small intestine length, the weight of small intestine, wet content (digesta) weight, and microbial mass pallet weight in phenobarbital-treated and control groups were indistinguishable, emphasizing the robustness of the results. Upon thorough analysis of results, it was observed that microbiome absorbed the phenobarbital in a specific pattern, showing low absorbance at transit time of 3 h, continued to plateau at 4 h, decreased to a minimum at 5 h and vanished completely at a transit time of 6 h depicting a state of competitive absorbance between the microbiome and the epithelial cells (enterocytes) in the small intestine. The maximum concentration of phenobarbital was absorbed by the gut microbiome at transit time of 4 h and continued till 5 h post drug administration confirming a maximum transit time of 5 h for orally administered drugs.

According to literature archive, phenobarbital is absorbed by mode of passive transport as it is lipophilic and weakly acidic in its chemical nature [30]. Phenobarbital bears molecular weight of 232.24 with one peptide bond in its structure (Fig. 1), and a size of dipeptide that suggests the involvement of POT in the uptake of phenobarbital. On this structural basis, phenobarbital presents itself as a potential substrate of the gut microbiome as absorbed through YdgR (POT) transporter present in the genome of gram-positive [31] as well as gram-negative gut microbes [22] as shown in protein-phenobarbital interaction through docking studies (Fig. 7A & B). Pockets in the cell membrane exhibit hydrogen bonding that facilitate the uptake of phenobarbital inside the cell membrane of microbial cells residing in the gut as percent dose recovery 5.73% which is a possible answer of missing oral dose of phenobarbital while bioavailability of phenobarbital is 94.9% [32]. Recent studies confirmed that the microbiome absorbed paracetamol (13.16%) and sulpiride (3.91%) at a transit time of 4 h respectively [23, 24] while phenobarbital at a dose of 15 mg/kgbwt [25] when administered orally was absorbed up to 5.73 ± 0.19% at 4 h. In the current docking study, weak interaction through less H bond within the active site of the transport protein (PDB ID: 5A9D) protein as well as the membrane protein (PDB ID: 6GS4) suggests phenobarbital as a poor substrate (Fig. 7C & D) of the gut microbiome showing surface binding (Fig. 7E & F) interactions. That was agreed with the in vivo trial which possesses the lowest dose recovery. So, the maximum drug was absorbed by passive transport [30]. Previously, in in vitro study, using multiple drugs [22] have also reported the absorbance of different drugs through YdgR overexpression and normal *E. coli* BL21 strains. Microbiome absorbed variable concentrations of phenobarbital at different time intervals advocating a competitive state with enterocytes lining the small intestine. Therefore, it can be hypothesized that the phenobarbital absorbed by the intestinal microbiome in a non-linear pattern may be either metabolized inside the microbial cell or extruded through efflux transporters by primary active transporters [14] as a limitation of the current study. Clinically, the current ‘in vivo microbial drug absorbance assay’ is worthy for expensive exogenous compounds such as anticancer drugs to save their quantity, if absorbed, by inhibiting the microbial absorption through the development of new entities. The current study also establishes the need to explore the potential of other orally administered drugs for their microbial absorbance in the gut.

## Conclusions

Orally administered non-antibacterial phenobarbital is absorbed by the intestinal microbiome through primary and secondary transport mechanisms thus confirming the existence of structural homology in transport mechanisms in microbial cell membranes and apical membrane of enterocytes. Through discovering new entities, strategies should be adapted to enhance the bioavailability of orally administered drugs by inhibiting the drug absorbance by microbial cells. This study is thought-provoking for pharma legislative authorities to have a better understanding of the drug-microbiome interactions to revisit each drug’s monograph by addition of ‘in vivo microbial drug absorbance assay’, not addressed so far.

## Data Availability

All data generated or analysed during this study are included in this published article.
